# The annulus: composition, role and importance in sperm flagellum biogenesis and male fertility

**DOI:** 10.1186/s12610-024-00241-2

**Published:** 2024-12-16

**Authors:** Marjorie Whitfield

**Affiliations:** Institute for Advanced Biosciences, INSERM U 1209, CNRS UMR 5309, Université Grenoble Alpes, Team ‘Physiopathology and Pathophysiology of Sperm cells’, 38000 Grenoble, France

**Keywords:** Sperm, Annulus, Flagellum, Spermiogenesis, Septin, Capacitation, Fertility, Spermatozoïde, Annulus, Flagelle, Spermiogenèse, Septin, Capacitation, Fertilité

## Abstract

The annulus is an electron-dense ring structure that surrounds the axoneme and compartmentalizes the sperm flagellum into two parts: the midpiece and the principal piece. The function of the annulus as a diffusion barrier in the mature spermatozoon is now well described but its function during spermiogenesis remains unclear. The intriguing spatio-temporal dynamics of the annulus during spermiogenesis and its position at the interface of the two main flagellar compartments have been highlighted for more than 50 years, and suggest a major role in this process. During the last decade, numerous studies contributed in establishing a repertoire of proteins known to be located at the annulus. Mutant mouse models of invalidation of these proteins have provided essential information and clues for novel hypotheses regarding the functions and regulation of this structure. Importantly, the recent identification in humans of homozygous mutations of genes coding for annulus proteins and leading to sterility have reinforced the importance of this ring structure for sperm physiology and male fertility. This review provides a comprehensive description of all the knowledge obtained in the last several years regarding the annulus composition and functions, both in mice and in humans.

## Résumé

L'annulus est une structure en anneau qui entoure l'axonème et compartimente le flagelle du spermatozoïde en deux parties : la pièce intermédiaire et la pièce principale. La fonction de l'annulus comme barrière de diffusion dans le spermatozoïde mature est maintenant bien établie mais sa fonction au cours de la spermiogenèse reste inconnue. La dynamique spatio-temporelle de l’annulus au cours de la spermiogenèse et sa position à l’interface des deux principaux compartiments flagellaires ont été mises en évidence depuis plus de 50 ans et suggèrent un rôle majeur dans ce processus. Au cours de la dernière décennie, de nombreuses études ont permis d'élargir nos connaissances sur le répertoire de protéines présentes au niveau de l'annulus. Les modèles de souris mutantes pour certaines de ces protéines ont fourni des informations essentielles et de nouvelles hypothèses concernant les fonctions et la régulation de l’annulus. De manière importante, l’identification récente, chez l’homme, de mutations homozygotes dans des gènes codant pour des protéines de l’annulus et conduisant à une infertilité, a renforcé l’hypothèse d’un rôle essentiel de cette structure pour la physiologie des spermatozoïdes. Cette revue fournit une description complète des connaissances acquises au cours des dernières années concernant la composition et les fonctions de l'annulus chez la souris et chez l'homme.

## Introduction

**Fig. 1:**
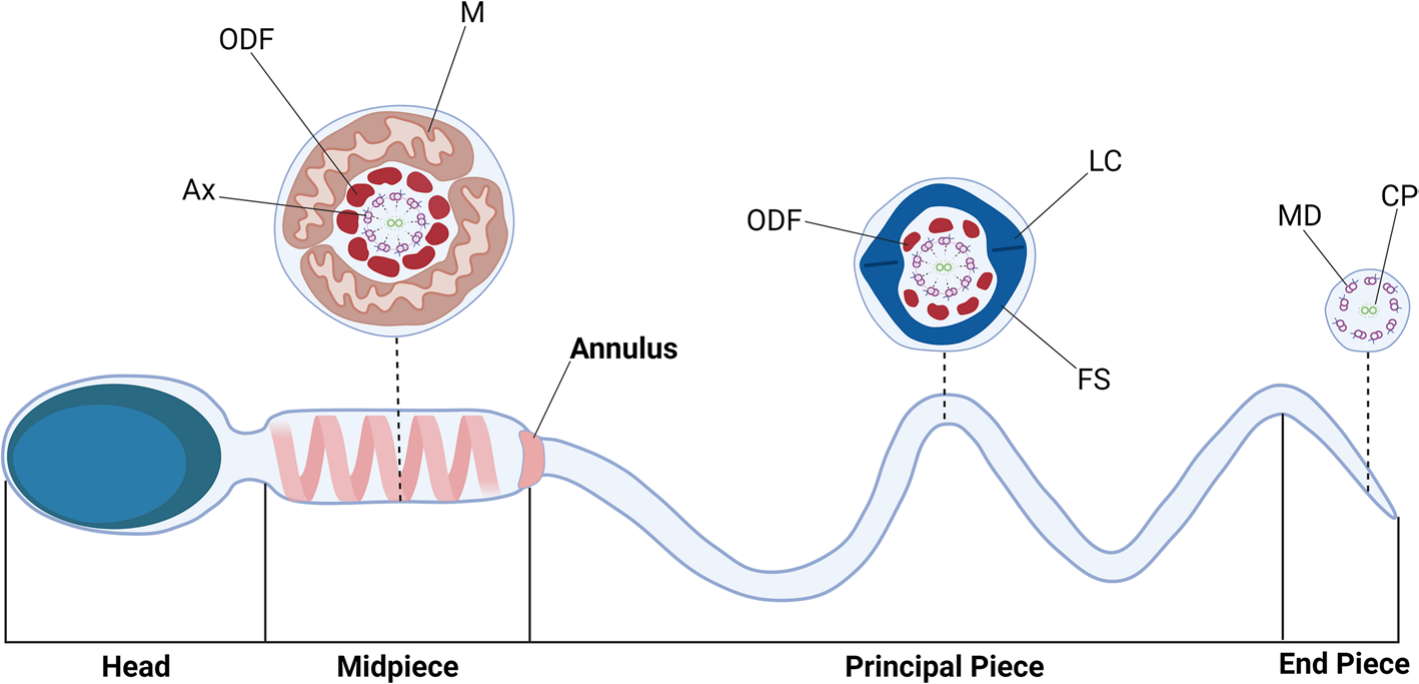
Schematic representation of the spermatozoa with cross sections of the different segments. The flagellum is composed of a central structure called the axoneme, a structure composed of 9 peripheral microtubules doublets and a central pair. The flagellum is divided into two parts: the midpiece which contains the mitochondrial sheath and the dense fibers, surrounding the axoneme, and the principal piece which contains the fibrous sheath around the axoneme. These two main compartments are separated by the annulus. The terminal piece of the flagellum only contains the axoneme. Ax: axoneme, ODF: Outer Dense Fiber, M: Mitochondria, LC: Longitudinal columns, FS: Fibrous Sheath, MD: Microtubule doublet, CP: Central pair. Original figure, created with BioRender.com

**Fig. 2:**
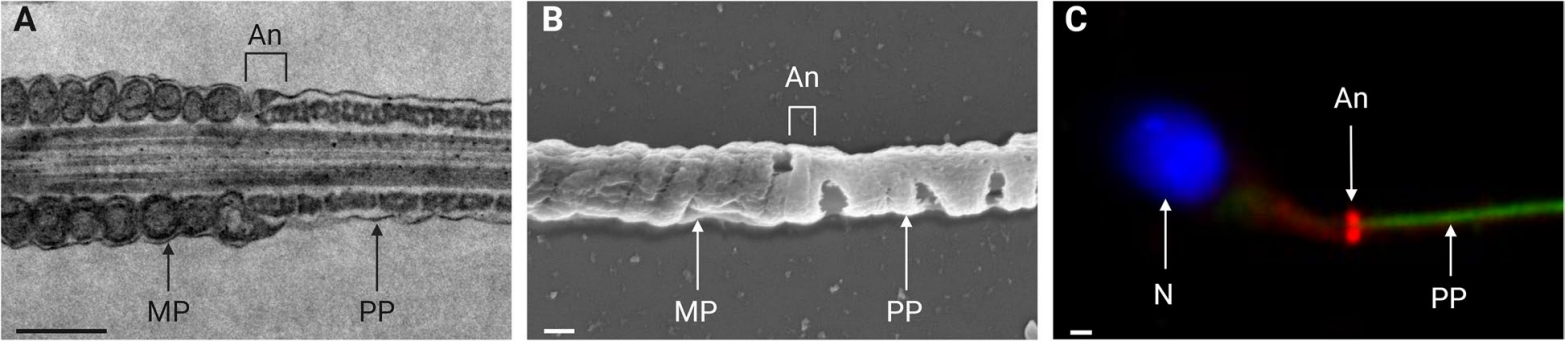
Annulus microscopy. **A** Longitudinal section of mouse flagellum in transmission electron microscopy. Scale bar: 500nm. **B** Mouse flagellum in scanning electron microscopy. Scale bar: 200nm. **C** Immunofluorescence detection of the annulus in human sperm (SEPT4 in red, principal piece in green, DNA in blue). Scale bar: 1µm. An: Annulus, MP: Midpiece, PP: Principal Piece, N: Nucleus. Original figure

Spermatogenesis is the long, complex, multi-step process by which spermatozoa are produced in the testis before being matured during epididymal transit. It begins with the proliferation of spermatogonial stem cells, which is followed by the meiotic division of spermatocytes and morphological differentiation of spermatids. This last step — spermiogenesis — mediates the transition from a round spermatid to the elongated and highly differentiated spermatozoon. The structure of the spermatozoon consists of two main parts —a head capped with the acrosome, and a flagellum or tail including a microtubule-based structure, the axoneme. The tail consists of two main compartments: the midpiece (MP), which contains the mitochondrial sheath and the principal piece (PP) which contains the fibrous sheath. The terminal piece of the flagellum solely harbors axoneme. The MP and PP are separated by the annulus, also known as Jensen's ring (Fig. 1). Under a transmission electron microscope (TEM), the annulus can be visualized as an electron-dense ring-shaped structure firmly attached to the plasma membrane. In immunofluorescence microscopy, this structure appears as two well-defined dots at the MP/PP junction (Fig. 2).

**Fig. 3:**
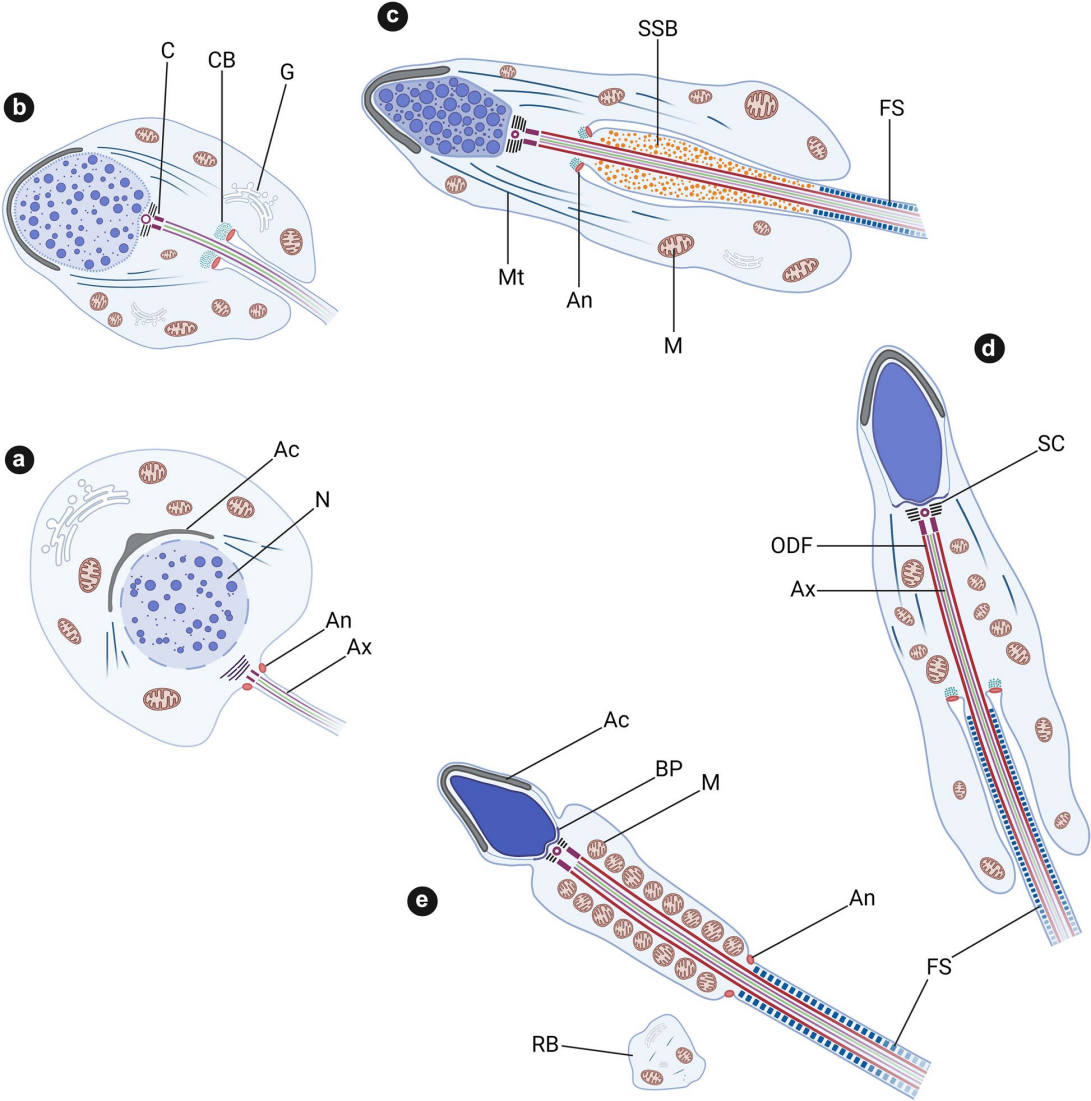
Schematic representation of sperm flagellum biogenesis during spermiogenesis. During spermiogenesis (see steps 1 to 5), the sperm cell undergoes a set of morphological modifications allowing the transformation of a round spermatid to an elongated spermatid, and ultimately, a spermatozoon. The fusion of the Golgi vesicles allows the formation of the acrosome, and the compaction of the DNA by protamines leads to the nucleus condensation. At the same time, the flagellum initiates its growth with the assembly of the axoneme from the centriole, and positioning of the peri-axonemal structures (**a**). During flagellar growth the annulus slides along the axoneme, accompanied by the chromatoid body (**b**–**d**). Upstream, the mitochondria are arranged in a mitochondrial sheath. Then the annulus stops at its final position between the midpiece and the principal piece (**e**). The excess of cytoplasm and organelles are eliminated into the residual body (**e**). Ac: Acrosome, N: Nucleus, An: Annulus, Ax: Axoneme, C: Centriole, CB: Chromatoid body, G: Golgi, Mt: Manchette, M: Mitochondria, SSB: Spindle Shaped Body, FS: Fibrous Sheath, SC: Segmented Column, ODF: Outer Dense Fiber, BP: Basal Plate, RB: Residual Body. Adapted from Holstein and Roosen-Runge 1981. Created with BioRender.com

The dynamics of the annulus during spermiogenesis are surprising. This structure appears at early stages of flagellum formation and is initially located at the base of the flagellum, close to the membrane and to the nucleus (Fig. 3a). During flagellum elongation, the annulus — still firmly attached to the plasma membrane — slides along the axoneme(Fig. 3b). During this migration, the annulus remains upstream from the mitochondria, which begin to arrange themselves around the dense fibers and the axoneme (Fig. 3b). The chromatoid body, a more diffuse structure composed of ribonucleoprotein granules, accompanies the movement of the annulus during spermiogenesis (Fig. 3b–d) but does not persist in the definitive spermatozoon. The ring then stops at its final position, separating the MP and the PP (Fig. 3e) [1, 2]. At the same time, the mitochondria condense and become organized into a double helix that forms the mitochondrial sheath within MP [3].

Little is known about the exact function of the annulus, particularly during spermiogenesis. The dynamics of its formation and migration suggest that this intriguing structure may be involved in the stages of flagellum biogenesis. Recent studies have shown that a deficiency of the proteins located at the annulus leads to infertility in both mice and humans. These findings suggest that this structure plays an essential role in sperm physiology and highlight the importance of defining its molecular composition and functions in more detail. In this review, the first comprehensive review on the subject since 2012, we will provide an overview of the current knowledge about the composition of the annulus. We will review in detail the characteristics of the proteins present at the annulus and the phenotype of mouse models in which the genes encoding these proteins are invalidated. We will then discuss the known roles and regulation of this structure and propose new hypotheses concerning its function. Finally, we will present the phenotypes observed in humans with mutations of annulus protein-coding genes and a comparison of these phenotypes with those observed in mice.

## Overview of the sperm annulus composition

Studies by several different teams led to the identification of a dozen of proteins that are present in the annulus. These proteins belong to different classes ranging from proteins with structural properties, ion channel, chaperone to mitochondrial and cytoplasmic enzymes; some of them being constitutively present in the ring whereas others are only transiently present.

Overall, from these studies, it is established that the main architecture of the ring is created by proteins with a mostly structural role, such as the septins (SEPT proteins), which are organized into complex octamers to form the ring core. Septins 1, 2, 4, 6, 7, 10, 11, 12 are all present in the annulus [4–7]. The transmembrane anion transporter SLC26A8 (TAT1) also seems to be involved in the anchoring of the annulus to the membrane [8]. The CBY3 and ciBAR1 proteins are present in the migrating annulus and seem to be involved in its correct positioning [9]. The cochaperone DNAJB13 is also transiently present at the annulus during spermiogenesis [10]. Several enzymes, such as CYP24A1 [11] and the soluble adenylate cyclase (SACY), are also found in the annulus [12]. Androglobin, a globulin family protein with a protease domain, is also found in the annulus from spermatid stage 15 onwards [13].

Other proteins at the annulus have been reported without providing firm evidence to confirm their presence in this structure, such as co-staining by immunofluorescence with known annulus proteins. This is the case for MgcRabGAP [14], DDX6 [15], AGTPBP1 [16], REEP6 [17] and ALF [18].

In the following chapter, we will provide a comprehensive description of all proteins reported to date, as components of the sperm annulus, and discuss how they contribute in conferring proper sperm structure and function.

## Detailed description of the sperm annulus components

The components of the annulus have mostly been studied in mice by means of genetically modified mice (knockout – KO and knock-in– KI models), which have provided valuable information. Hence, studies in these models have demonstrated the importance of the annulus for reproduction as all these mutant mouse models display fertility defects. Below, we describe the known functions of annulus proteins and the associated sperm phenotypes in KO models; the details of the mouse models’ phenotypes are summarized in Table 1.

**Table 1:** List and description of mouse models genetically modified for a gene encoding an annulus protein

Protein (location)	Article	Genetic Model	Fertility	Sperm count	Phenotype of spermatozoa	Annulus phenotype/barrier	Other annulus proteins	Capacitation	ATP
**SEPT4** (annulus)	[6, 20]	Knock out (loss of exons 2–12)	Sterile	Normal	Spz bending at MP/PP junction mitochondria abnormalities	Absent, membrane diffusion barrier altered	(Not specified)	Absence of Tyrosine phosphorylation	(Not specified)
	[4]	Knock out (loss of exons 2–10)	Sterile	Normal	Spz bending at MP/PP	Absent	SEPT absent at the annulus but normal levels in WB for 1, 2, 6, 7, 11	(Not specified)	ATP produced but lack of consumption
**SEPT12** (neck, midpiece, annulus)	[21]	Chimeric knock out in ES cell (loss exon 3 to 7)	Variable depending on the chimerism degree	Decreased sperm count	Variable depending on the chimerism degree spz bending, disorganized mitochondria, abnormal nuclei and acrosomes	(Not specified)	(Not specified)	(Not specified)	(Not specified)
	[5]	SEPT12^D197N^ Mutation in GBD domain	Sterile	Normal	Spz bending	Absence of SEPT polymer at the annulus but no TEM	SEPT2, 6, 7 lost at the annulus	(Not specified)	(Not specified)
	[23, 24]	SEPT12^S196E^ Phosphomimetic	Infertility	Decreased sperm count	Spz bending	Absent	SEPT4 and 12 lost at the annulus but normal level in testis in WB for SEPT2, 4, 6, 7, 12	Hyperactive motility and tyrosine phosphorylation decreased	No alteration in cellular model
	[24]	SEPT12^S196A^ phosphodeficient	Fertile in vivo Fertility decreased in IVF	Normal	Normal	Normal	All SEPT are present at the annulus	Hyperactive motility and tyrosine phosphorylation slightly decreased	No alteration in cellular model
**SLC26A8** (annulus)	[8]	Knock out (loss of exon 2)	Sterile	Normal	Spz bending at MP/PP junction mitochondria abnormalities	Present but abnormal (abnormally shaped, detached for the membrane)	SEPT4 present at the annulus	Absence of Tyrosine phosphorylation	ATP produced but lack of consumption
**CBY3** (distal appendage at S9, S15 at the annulus, loss at S16)	[9]	Knock out (loss of exon 2)	Sterile	Decreased sperm count	Spz bending	Present but mispositioned into the principal piece membrane diffusion barrier altered	SEPT4 present at the annulus	Hyperactive motility and tyrosine phosphorylation decreased	(Not specified)
**ciBAR1** (distal appendage prior to S9, S15 at the annulus, loss at S16)	[9]	Knock out (loss of exon 2)	Infertility (70% reduction in litter size)	Decreased sperm count	Spz bending	Present but mispositioned into the principal piece membrane diffusion barrier altered	SEPT4 present at the annulus	(Not specified)	(Not specified)
**Androglobin** (neck, flagellum, annulus at S15 and mature sperm)	[13]	Knock out (loss of exon 13–14)	Sterile	Testis weight reduced, presence of post-meiotic events but few flagella	Almost absence of mature spz, abnormally shaped heads	**-**	Septs expressed in the testis	**-**	**-**

For each mouse model, the fertility status, the state of spermatogenesis, the sperm phenotype and in particular at the annulus is given. When the information is known, the maintenance or not of septins at the annulus is given, as well as the ability to carry out capacitation and produce ATP (ATP was measured when the spz were sampled and after one hour of incubation, either by luciferase method^4^ or via a luminescent kit^8^)

"S9,15,16" indicate the different stages of spermiogenesis in mouse

*ES* Embryonic Stem, *IVF* In Vitro Fertilization, *MP* Midpiece, *PP* Principal Piece, *TEM* Transmission Electron Microscopy, *WB* Western Blot, *Spz* Spermatozoa, **-**: irrelevant

### Septin proteins

The septin proteins are GTPases that have been conserved throughout evolution. They self-assemble into a core hexamer or octamer via their GTP-binding domain (GBD) and an NC (N- and C-termini) interface and can form filaments or rings, which are involved in many cellular processes. There are 13 functional septin genes in humans (*SEPT1* to *SEPT12* and *SEPT14*), classified into four subgroups on the basis of sequence similarity: the SEPT2 subgroup (containing SEPT1, 2, 4 and 5), the SEPT3 subgroup (SEPT3, 9 and 12), the SEPT6 subgroup (SEPT6, 8, 10, 11 and 14) and the SEPT7 subgroup (containing only SEPT7) [19]. SEPT12 is expressed specifically in the testis and studies in cellular models have shown that this protein organizes the core of the annulus into octameric septin filaments. These octamers are formed with a precise sequence, with SEPT12 molecules at each end: SEPT12-7–6-2–2-6–7-12 or 12–7–6–4-4–6-7–12 [5]. The SEPT12 proteins of different octamers connect with each other via the NC interface, whereas the other SEPT proteins within the octamer bind via their NC or GBD domains. SEPT2 and SEPT4, both of which belong to the SEPT2 subgroup, appear to be interchangeable within the complex. This octamer sequence has been demonstrated only in cellular models, but it is highly likely that a similar sequence occurs in the base of the sperm annulus in vivo. Immunostaining has revealed the likely presence of other septins at the sperm annulus: SEPT1 [4], SEPT10 [7, 13] and SEPT11 [7]. These SEPTs may replace a septin from the same subgroup in the octamer. Septins have also been detected elsewhere in spermatozoa (head, neck, MP), suggesting that they probably also have roles outside the annulus.

Genetically modified mouse models have played a particularly important part in highlighting the role of septins. One of the first such models was the *Sept4* KO mouse, in which *Sept4* was deleted from the mouse germline, eliminating all transcripts generated from this locus throughout the entire body of the offspring [4, 6, 20]. Homozygous mutant mice were viable, with no visible abnormalities. However, KO males were sterile, with significant defects of the spermatozoa. The histology of the testicles was normal in KO mice, but the spermatozoa in the cauda epididymis were entirely non-motile and 50 to 70% of them displayed very strong bending, frequently at 180° to form a "hairpin" at the junction of the MP and PP. The authors investigated the origin of this phenotype by studying the morphology of spermatozoa in the testis and caput epididymis. The sperm had thin flagella sections, in place of the annulus but 180° folding had not yet occurred, suggesting that this defect appear at late stages of spermatogenesis, with folding occurring even later, due to mechanical constraints during epididymal transit. The site at which folding occurred suggested an annulus deficiency, which was confirmed by transmission electron microscopy (TEM). Indeed, the annulus was completely absent in the mutant sperm, with no electron-dense structure visible. The region immediately following the junction between the MP and PP had no fibrous sheath. Moreover, the mitochondria in wild-type sperm are normally highly homogeneous and ordered along the axoneme in the MP, whereas they were variable in size and appearance in Sept4 KO sperm (fewer cristae, less membrane material, fission). The SEPT4 is, therefore, clearly important for the architectural organization of the flagellum.

SEPT12 (the testis-specific septin) has also been studied in detail through the creation of numerous mouse models. The level of *Sept12* expression was shown to be crucial for spermiogenesis. Studies of chimeric mutant mice with a partial invalidation of *Sept12* revealed that the impact on sperm morphology was correlated with the degree of chimerism. The spermatozoa presented abnormalities of the acrosome, head and flagellum, again with a large proportion of bent or folded spermatozoa [21]. Another mouse model with a mutation affecting the GBD domain of *Sept12* [5], the D197N mutation, produced sperm with significant morphological defects, including a bent tail, resulting in a significant decrease in motility. This mutation disrupts the formation of SEPT12 filaments by preventing the binding of SEPT12 to SEPT7 via the GBD domain. This leads to the loss of septins 12, 7, 6 and 2 from the annulus. These findings demonstrate the importance of the GBD domain for the formation of septin octamers and, therefore, for formation of the annulus.

The importance of post-translational modifications (PTM) to septins for the dynamics of septin filaments has been clearly demonstrated in yeast [22]. A phosphomimetic SEPT12 KI model was created in mice, to investigate the regulation of septins by PTM in spermatozoa. The serine residue in position 196, which is highly conserved between organisms (corresponding to position 198 in humans), was mutated to a glutamate residue to mimic constitutive phosphorylation [23, 24]. The results obtained with this model suggested that constitutive phosphorylation prevents formation of the septin polymer and, thus, of the annulus. These mice had normal reproductive organs and sperm counts but their fertility was very low. Indeed, more than 80% of the spermatozoa presented morphological defects, with a thinning of the annulus region often resulting in flagellum folding, as described above. We will return later to the role of septin phosphorylation in the regulation of the annulus.

### SLC26A8

The Testis Anion transporter 1 (TAT1 or SLC26A8) is specifically and highly expressed at the spermatocyte stage [25, 26]. SLC26A8 is an integral membrane protein with anion transporter activity in vitro that cooperates with the CFTR channel to regulate the Cl^−^/HCO_3_^−^ fluxes required for motility and capacitation [25, 27]. SLC26A8 localizes to the annulus of sperm, both in mice and in humans [8]. In mouse, *Slc26a8* invalidation also leads to the characteristic folding of the flagellum at the MP/PP junction. While, in *Slc26a8*-null sperm, the annulus was shown to be present, it was reported with an abnormal oval shape and ectopic location or sometimes, isolated from other flagellar structures [8]. These data suggest that SLC26A8 may be involved in anchoring the annulus to the plasma membrane. Thus, despite the presence of a partial annulus structure in this model, the fragility of the MP/PP junction indicates that it is probably unable to fulfill its function. The sperm mitochondria also appear to be affected in this model, with the MP displaying a slight disorganization and containing mitochondria of heterogeneous sizes [8].

### Androglobin (Adgb)

Androglobin, a member of the globin family, was recently shown to be expressed at late stages of spermatogenesis [13]. This protein of unknown function contains an N-terminal calpain-like protease domain, a central globin domain with a calmodulin (CaM)-binding IQ motif and a C-terminal coiled-coiled region [28]. It is present in the neck and flagellum, particularly the annulus of stage 15 spermatids and mature spermatozoa. Androglobin has been colocalized with SEPT10 at the neck and annulus and it has been suggested that it participates in annulus regulation through CaM-dependent proteolysis [13]. In mice, *Adgb* invalidation leads to infertility with severe defects of spermatogenesis. Post-meiotic events occur in this model, but flagella are rarely. Spermiation also seems to be impaired, contributing to severe oligozoospermia. When the flagella are visible, they present major defects, as they are short, thin, coiled or irregular, with defects in the mitochondrial sheath. Moreover, heads of the sperm are also abnormally shaped [13, 29]. Androglobin may be involved in maintaining the annulus/flagellum structure, but it is difficult to draw any firm conclusions about possible annulus abnormalities in these conditions. Additional studies are, therefore, required, to determine the role of ADGB.

### CYP24A1

CYP24A1 (1,25-dihydroxyvitamin D(3) 24-hydroxylase) is an enzyme that degrades the physiologically active form of vitamin D. It is normally found in the mitochondria of somatic cells [30]. CYP24A1 was also detected in the annulus of human sperm, and this localization was confirmed by double-labeling for SEPT4. However, only a small proportion of the SEPT4-positive spermatozoa were double-labeled for CYP24A1. Overall, the role of this protein in the annulus remains unclear, but the results of this study suggest that its presence is associated with better sperm quality [11]. It is also possible that this protein is not always present at the annulus. Indeed, the proteins which will be described below are known to be transiently localized at the annulus.

### DNAJB13

DNAJB13 is a type II HSP40/DnaJ protein from the HSP40 cochaperone family [31]. The *DNAJB13* gene is expressed specifically in the testis in mice, and the encoded protein is found in the cytoplasm of spermatids and associated with the axoneme of the sperm flagellum [31]. Double-labeling with an anti-SEPT4 antibody revealed that DNAJB13 was present in the annulus, only transiently during spermiogenesis [10]. The DNAJB13 was first detected in the annulus at the appearance of this structure in stage 9 spermatids. The signal increased during spermiogenesis, but then gradually disappeared from the junction between the MP and PP of mature sperm, persisting in the axoneme within the flagellum, probably in the radial spokes [10, 31, 32]. Mice with an invalidated *Dnajb13* gene develop hydrocephalus leading to their death before sexual maturation, precluding studies of their reproductive phenotype. This problem was resolved by the creation of chimeric mice with biallelic mutation in embryonic stem cells. The spermatozoa of these mice displayed severe morphological defects of the flagellum [33]. However, as DNAJB13 is localized in the radial spokes of the mature spermatozoon, it was not possible to distinguish between the axonemal role of this protein and its role in the annulus during spermiogenesis.

### Soluble adenylyl cyclase

**Fig. 4:**
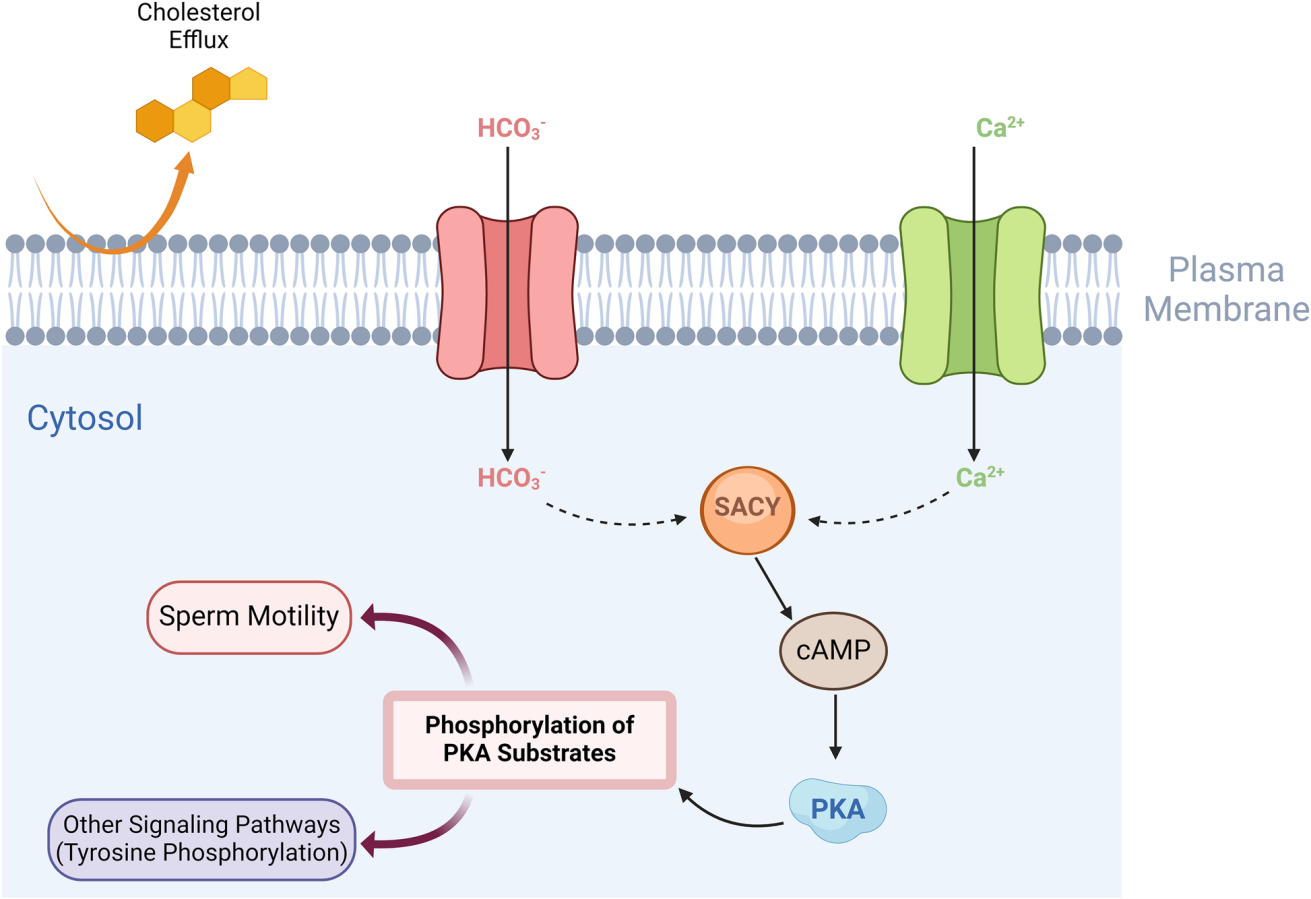
Schematic representation of the main events occurring during sperm capacitation. Capacitation is the functional maturation event occurring during sperm transit through the female reproductive tract. It involves a set of biochemical and functional modifications, which are essential to confer the fertilization potential. Overall, the process begins with an efflux of cholesterol from the sperm membrane and an influx of calcium and bicarbonate ions, into the spermatozoon. These modifications lead to the activation of the soluble adenylate cyclase (SACY), which produces cAMP that directly activates the protein kinase A (PKA), resulting in an increase in protein tyrosine phosphorylation inside the spermatozoon. Created with BioRender.com

The second messenger cAMP regulates essential physiological processes in the spermatozoon. It is produced by an atypical soluble adenylate cyclase: SACY (or sAC). This enzyme is activated by bicarbonate and calcium, particularly during capacitation, which occurs in the female genital tract and is essential for fertilization (Fig. 4) [34]. SACY has a punctate distribution along the midpiece and at the annulus in non-capacitating sperm. However, following capacitation, the majority of SACY is concentrated very strongly to the annulus [12], suggesting a role of the annulus in the capacitation process (see section New insights into the functions of the annulus).

### The Cby3/ciBAR1 complex

The coiled-coiled protein Chibby 3 (Cby3) is expressed exclusively in the testis and in elongating spermatids in particular. It is located at the flagellar base, very close to the annulus, at stage 9 (S9) of spermiogenesis. It accompanies the migration of the annulus during S14 and is found at the MP/PP junction at S15, colocalized with SEPT4. Cby3 disappears at S16 and is absent from mature spermatozoa. The ciBAR1 protein (for Cby1-interacting BAR domain-containing 1) is present slightly earlier than Cby3, at the round spermatid stage. It then follows the same path as Cby3, and is absent from mature spermatozoa. Ultrastructure expansion microscopy has revealed that CBY3 and ciBAR1 form a ring-like structure within the septin ring. These data provide valuable information about annulus architecture during spermiogenesis [9]. Interestingly, the invalidation of *Cby3* or *Cibar1* results in sharply bent flagella, as in models presenting annulus defects. However, the annulus is not lost in this case, but present at an abnormal location in the principal piece. These two proteins were found to function as a complex in the compartmentalization of the sperm flagellum. Moreover, this complex was found to be essential for controlling the precise location along the flagellum at which the annulus ceases its migration [9].

Altogether, the studies performed so far in mouse models have contributed in establishing a small but much valuable repertory of the components of the sperm annulus. These studies indicate that these components, transiently or constitutively present, are essential for proper annulus integrity, flagellum structure and male fertility, although the associated molecular mechanisms remain unclear.

## Role and regulation of the annulus

In this chapter, we will further discuss the data obtained from mutant mouse models, which have shed some light on the potential functions of the annulus, in particular during spermiogenesis.

### Structural properties

The various mutant mouse models of genes encoding annulus proteins have highlighted a common phenotype characteristic of alterations to this structure: bending of the flagellum at the MP/PP junction. The presence of an annulus is not sufficient to ensure that this structure is effective. Indeed, a ring is clearly visible in the *Slc26a8* and *Cby3*/*Cibar1* KO models (unlike septin mutant models) and yet folding is nevertheless observable [8, 9]. Indeed, in these models, the annulus is either detached from the membrane (*Slc26a8*^*−/−*^) or located in the PP (*Cby3*^*−/−*^, *Cibar1*^*−/−*^). An absent or poorly positioned/malformed annulus therefore systematically leads to a characteristic hairpin flagellum phenotype (mutation of *Sept4, Slc26A8, Sept12, Cby3, Cibar1*) due to the fragility of the structure.

The structural role of the annulus does not seem to be restricted to the MP/PP junction. Indeed, in several previously described models, the organization and structure of the mitochondria were found to be altered. This phenotype was initially reported in the *Sept4* KO model, leading to suggestions that proper mitochondria organization might be dependent on SEPT4 rather than the annulus. Indeed, SEPT12 KI models do not present any mitochondrial defect despite the absence of the annulus. In addition, certain isoforms of SEPT4, such as ARTS, are known to be mitochondrial [35], leading to suggestions of a role of these specific isoforms within mitochondria. However, there are published data refuting this hypothesis. For example, mitochondrial abnormalities have been reported in a model of chimeras with *Sept12* invalidation [21]. The mitochondria in this model appeared to be disorganized, with an abnormal content. Furthermore, sperm mitochondrial also appeared to be affected in a mouse model of *Slc26a8* invalidation, (sheath disorganization and mitochondria of heterogenous size -[8]), resulting in a phenotype similar to that of the *Sept4*^*−/−*^ model. In addition, no SEPT4 immunofluorescence was detected in the MP.

Taken together, these data strongly support a link between the annulus and the mitochondrial architecture but further studies will be required to characterize the mitochondrial abnormalities and this link in more detail. To note, it is regularly reported that the annulus participates in the establishment of the mitochondrial sheath during spermiogenesis. For example, Shen et al. (2017) described one of the roles of the annulus as the “establishment of the mitochondrial distribution” [23]. However, the mitochondrial sheath generally forms in the absence of the annulus and the mitochondria are not distributed randomly throughout the sperm [6, 8]. This interpretation, thus, remains a matter of conjecture based on the concomitant migration of the annulus and the mitochondria. In the mouse models described above, it is not known whether the mitochondria are affected early on and additional studies are required to determine the precise role of the annulus during spermiogenesis.

In conclusion, we can conclude that the annulus has a structural role because it participates in the definition of the two flagellar compartments (i.e. MP and PP), but this barrier is not only physical it is also functional.

### Selective diffusion barrier

Certain proteins, although capable of diffusing freely within the plasma membrane, are restricted to specific flagellar compartments [36]. This is the case, for example for basigin. This transmembrane glycoprotein is located in the PP in caput epididymal in rats and mice spermatozoa. During epididymal transit, this protein relocalizes to the MP [37].

The *Sept4* invalidation model provided clues to the mechanisms by which such proteins are regionalized with the flagellum. Indeed, in spermatozoa from *Sept4* mutant mice, basigin has a diffuse distribution along the length of the flagellum and even in the sperm head, at all stages of maturation [20]. Similar observations were made in the *Cby3* and *ciBAR* KO models [9]. All these data indicate that the annulus plays a direct role in restricting the diffusion of membrane proteins within the sperm. Furthermore, this role as a diffusion barrier appears to be modular because specific proteins are translocated at specific times. In guinea pig, the PT-1 protein remains restricted to the PP in the mature sperm in the cauda epididymis, contrasting with observations for basigin. PT-1 crosses the annulus barrier only at the time of capacitation [38]. Unfortunately, it has not yet been possible to localize PT-1 in the sperm of *Sept4*^*−/−*^ mice due to the lack of a functional antibody.

It remains unclear how the annulus acts as a barrier. Freeze-fracture electron microscopy analysis of the membrane above the annulus has shown this membrane to be rich in particles organized into a dense circumferential arrangement [39]. These particles may be transmembrane proteins docked at the annulus and acting as a veritable “fence”, regulating protein trafficking. In addition, the annulus is also a site with specific lipid territories. In particular, the membrane around the annulus is enriched in ganglioside M1 [40]. Local lipid composition may also, therefore, be involved in the establishment of a barrier to diffusion.

### Regulation of the annulus

If the diffusion barrier at the annulus is adjustable then it is very likely that this structure can be modified and respond to external stimuli. In this line, information regarding the molecular mechanisms that could regulate the function of the annulus as a diffusion barrier was mainly obtained through the studies of septin proteins.

As described above, the septin proteins are subjected to PTM, which participate in the regulation of septin filament dynamics in yeast [22]. PTM are also important for sperm annulus, as shown by studies of mice with a phosphomimetic mutation of *Sept12* [23], which have spermatozoa lacking an annulus (see section Detailed description of the sperm annulus components and Role and regulation of the annulus). Immunofluorescence analyses performed during spermiogenesis have shown that no annulus is formed despite the presence of all septins in normal amounts. Indeed, phosphorylation of the serine 196 residue of SEPT12 in these mice interfered with the SEPT12/SEPT7 interaction, inhibiting the association between SEPT12 and the SEPT7-6–6-2–2-6–7 complex. These studies demonstrated that the regulation of septins by phosphorylation is essential for the formation of the annulus.

Septin phosphorylation can also regulate the maintenance of the annulus during its passage through the epididymis. During transit, the epididymis sends the spermatozoa Wnt signals via its epididymosomes (small lipid vesicles). These signals block the activity of the GSK3 kinase, decreasing the phosphorylation of its targets, including SEPT4. In the absence of phosphorylation, septin polymerization persists, strengthening the diffusion barrier function [41]. The knockout of Ccnyl1 (*Ccnyl1*^*−/−*^ mice), a regulator of Wnt, in mice prevents the inhibition of GSK, leading to the phosphorylation of SEPT4. This phosphorylation disrupts the polymerization of septins at the annulus. Accordingly, the *Ccnyl1* mutant has a phenotype similar to that described for septin knockout, with characteristic bending at the annulus. The diffusion barrier is also lost, as demonstrated by the incorrect distribution of basigin in the cauda epididymis sperm cells (present throughout the flagellum instead of being restricted to the MP). Under normal conditions, basigin must pass through the annulus barrier during epididymal transit. This suggests that the permeability of the annulus must be modulated at this time point. The findings of this study suggest that this permeability may be regulated by the Wnt pathway and SEPT4 phosphorylation.

Overall, these data have established phosphorylation as a major regulatory mechanism of the annulus function; this point is consistent with the importance of protein phosphorylation events in the course of sperm motility and capacitation (Fig. 4). Some studies in yeast have identified other PTM, such as sumoylation and acetylation, which can modify the dynamics of septin polymerization [22, 42]. Further studies will be required to determine whether these PTM are also involved in mammals for the regulation of sperm annulus properties.

## New insights into the functions of the annulus

Several published findings indicate that the annulus may play a key role in sperm cell signaling, particularly during capacitation. Spermatozoa undergo capacitation — a set of biochemical and functional modifications in response to the microenvironment — within the female genital tract. They acquire the ability to fertilize the oocyte during this process [43].

The first major event in this process is the recruitment of SACY to the annulus (see section Overview of the sperm annulus composition) [12]. This enzyme is responsible of producing the cAMP required for the activation of PKA, a major kinase in the capacitation signaling cascade (Fig. 4) [34]. Its location at the annulus suggests a specific role for this region in the process of capacitation. As mentioned above, the plasma membrane in the annulus is locally enriched in GM1. This glycosphingolipid is known to be enriched in lipid rafts, veritable signaling platforms that bring together proteins essential for signaling pathways [44], providing further support for a role of the annulus in these processes.

SEPT12 is a direct substrate of the PKA, which is activated during capacitation [23], suggesting that the annulus may be regulated by septin phosphorylation during the process of capacitation. Murine models of the disruption of such phosphorylation are also informative. In addition to the phosphomimetic SEPT12 model described above, Wang et al. also reported a mouse model in which the Ser196 residue of SEPT12 could not be phosphorylated (*Sept12*^*S196A*^ – SA/SA model – 24). Unlike the other models described here, these mice presented no morphological alterations of sperm cells or modifications to the annulus. It was, therefore, possible to conclude that SEPT12 phosphorylation was not required for spermatogenesis and spermiogenesis. However, these mice displayed a clear change in in vitro capacitation, with both flagellar hyperactivation and changes in tyrosine phosphorylation levels (two markers of terminal capacitation Fig. 4). These findings indicate that the phosphorylation status of residue S196 of SEPT2 is important for capacitation. Moreover, SACY levels increase during capacitation, probably due to de novo translation. During capacitation in KI models, SACY was not recruited to the annulus in the SE/SE model and it was present in smaller amounts in both the SE/SE and SA/SA models. Thus, the timely phosphorylation of SEPT12 is required for SACY upregulation and recruitment to the sperm annulus. This finding also raises questions about the possible local translation of the enzyme in the annulus region. As mentioned in the introduction, the migrating annulus is accompanied by the chromatoid body during spermiogenesis. It is, therefore, possible that certain mRNAs remain localized in this region in the mature spermatozoon, but this remains to be demonstrated.

Furthermore, several mouse models with annulus defects presented an alteration of in vitro capacitation assessed by hyperactive motility and/or protein tyrosine phosphorylation levels [6, 8, 9, 23, 24].

Finally, the permeability of the barrier to diffusion created by the annulus is probably modified during capacitation by the passage of PT-1 from the PP to the MP, as shown in the guinea pig [38].

Ultimately all of these arguments suggest that the annulus plays a key role in capacitation, but it remains unclear how it participates in this process.

## Annulus defects in humans and comparison with mouse models

The significant abnormalities and impaired fertility encountered in mouse models with invalidation of genes encoding annulus proteins have led to investigations of the roles of these proteins in human infertility phenotypes; information about mutations identified in humans so far, has been summarized in Table 2.

**Table 2:** Genetic mutation of annulus protein-coding genes and associated phenotype of the spermatozoa in humans

Gene	Publication	Mutation	Results of semen analysis	Structural phenotype of spermatozoa	ART outcome
*SEPT4*	[57]	2 homozygotes patients c.A721T, p.R241* c.C205T, p.R69*	Asthenoteratozoospermia	Complete loss of annulus, thin midpiece, mitochondria were abnormal and desorganized. 20% bent sperm	Trial and successful ICSI for one couple
*SLC26A8*	[56]	2 heterozygotes bi-allelic patients (c.212G > T,p.Arg71Leu; c.290 T > C,p.Leu97Pro) (c.1664delT,p.Ile555Thrfs*11; c.290 T > C,p.Leu97Pro)	Asthenozoospermia	Complete loss of annulus, thin midpiece, very few mitochondria	Successful ICSI for both couples
*SLC26A8*	[54]	3 heterozygotes patients c.1570_1571del, p.A524* c.2191G > A, p.V731I c.306del, p.G103Afs*9	Asthenoteratozoospermia	No loss of SLC26A8 protein, no TEM of the annulus	No information
*SLC26A8*	[53]	3 heterozygotes patients c.260G > A, p.Arg87Gln c.2434G > A, p.Glu812Lys c.2860C > T, p.Arg954Cys	Asthenozoospermia	Decrease in SLC26A8 protein, impaired association with CFTR, no TEM of the annulus	No information
*SEPT12*	[5, 52]	2 heterozygotes patients c.266C > T, p.Thr89Met (T89M) c.589G > A, p.Asp197Asn (D197N)	T89M Asthenoteratozoospermia D197N Oligoasthenozoospermia	Complete loss of annulus for D197N and bent flagella	No information
*SEPT12*	[49]	Numerous SNP (cohort of 160 patients) Focus on c.474 A/A genotype, create a novel splice donnor site resulted in truncated protein	9 teratozoospermia 5 azoospermia	Bent tail, de-condensed nucleus, non-penetrant variant	No information
*SEPT12*	[65]	Some SNP were higher in SCOS patients cohort (100 patients)	Azoospermia	(Irrelevant)	No information

Description of *SEPT* or *SLC26A8* genetic mutations known in humans. Fertility status and sperm phenotype are given as well as ICSI outcome when known

*SNP* Single Nucleotide Polymorphisms, *TEM* Transmission Electron Microscopy, *ICSI* Intra Cytoplasmic Sperm Injection, *ART* Assisted Reproductive Technology

### Loss of the sperm annulus and human infertility

In an initial study, Sugino and collaborators have screened a cohort of 108 infertile patients and found that 13% presented abnormal sperm immunostaining for SEPT4, SEPT7, or both [45]. This suggested that annulus defects could lead to a significant proportion of asthenozoospermia. However, cohorts of asthenozoospermic patients have subsequently been screened for SLC26A8 immunostaining [46, 47] and the proportion of patients with defective immunostaining of the annulus was found very low (1/75 and 3/254, corresponding to 1.2% of the cohorts), suggesting that annulus abnormalities remain a rare event in human asthenozoospermia. An analysis of 100 asthenozoospermic patients performed by Hosseinifar and collaborators detected only one case of alterations to SEPT4/7 immunodetection, a finding more in line with those of the Touré laboratory cohorts [48]. This major difference between the prevalence of annulus defects in human asthenozoospermia may reflect the use of different proteins for the screening, and/or differences in patient selection, as the patients studied by Sugino were described as infertile but no details of their sperm characteristics were provided. Finally, ethnic differences between the different cohorts (Caucasian- Iranian—Japanese) may also contribute to these differences.

### SEPT12 mutations and human infertility

The first genetic studies in humans identified variants of the *SEPT12* gene. Exonic sequencing of *SEPT12* was then performed in large cohorts of fertile and infertile men, leading to the identification of many variants, including the c.474 G > A variant, which creates a novel splice donor site, leading to the loss of part of exon 5 [49]. Most individuals homozygous for this variant displayed teratozoospermia, with a significant proportion of bent flagella and decondensed nuclei, whereas others were azoospermic. These findings suggest an association with infertility, but this variant was also found in rare fertile patients, suggesting a potential incomplete penetrance, a well-known phenomenon observed for splice variants [50, 51]. Another study on a cohort of 160 infertile men identified two patients heterozygous for mutations of the *SEPT12* gene. One patient carrying the *c.266C* > *T*/p.Thr89Met mutation, which affects GTP hydrolysis activity, was asthenoteratozoospermic. The other, carrying the *c.589G* > *A*/p.Asp197Asn mutation, which affects GTP binding, was oligoasthenozoospermic [52]. These two variants were not present in fertile men. Finally, further investigation of the *SEPT12*^*D197N*^ patient confirmed the loss of the annulus, with an absence of SEPT12, 7, 6, 4 and 2 staining and evident folding of the flagellum [5, 52]. This phenotype is similar to that observed in mice carrying the same mutation. The mutant protein acting as a dominant negative, preventing the formation of the septin filament required for annulus assembly [5].

### SLC26A8 mutations and human infertility

Variants of the *SLC26A8* gene have also been identified. Two studies described infertile patients carrying heterozygous for mutations of this gene, but drew different conclusions. In an analysis of a cohort of 146 asthenozoospermic patients, Dirami et al. identified three patients heterozygous for nonsense mutations: *c.260G* > *A* (p.Arg87Gln), *c.2434G* > *A* (p.Glu812Lys), and *c.2860C* > *T* (p.Arg954Cys) [53]. In vitro analyses showed that the mutant proteins were able to bind the CFTR channel but unable to activate CFTR-dependent anion transport. We therefore assume that these variants acted via negative dominance, by binding CFTR without allowing its activation. These in vitro studies also showed that the mutant proteins were less stable, because they were more likely to be degraded via the proteasome. Consistent with this finding, the patients had lower levels of SLC26A8 protein. Some patients also had mitochondrial alterations and an incomplete annulus, a phenotype resembling *Slc26a8* knockout in mice [8]. In a more recent article, other mutations of the *SLC26A8* gene were detected at the heterozygous state in humans [54]. The three patients concerned presented three different mutations (*c.1570_1571del* [p.A524*]; *c.306del* [p.G103Afs*9]; *c.2191G* > *A* [p.V731I]) and displayed an asthenoteratozoospermia phenotype. Genetic analysis of the fathers of two of the patients showed that they also carried the genetic variant, raising doubts about the pathogenicity of the transmitted mutation. The authors concluded that heterozygosity for *SLC26A8* variants did not lead to infertility, specifying, in particular, that the diseases caused by mutations in SLC were all associated with a recessive mode of inheritance. However, heterozygosity for a mutation of the STAS domain of *SLC26A3* has already been shown to result in asthenozoospermia (SLC26A3-p.Asp688His). The molecular mechanism underlying male subfertility probably involves the dominant negative inhibition of CFTR by the SLC26A3 mutant [55]. These elements highlight the importance of taking into account the type of mutation. Different mutations of the same gene may have different effects, depending on the domains concerned, and this may even interfere with the mode of transmission.

Finally, patients with mutations of both *SLC26A8* alleles have also recently been identified; this is the case of two patients with compound heterozygous mutations (*c.290T* > *C*,p.Leu97Pro/ *c.1664delT*,p.Ile555Thrfs*11 and *c.290T* > *C*,p.Leu97Pro/*c.212G* > *T*,p.Arg71Leu) displaying severe asthenozoospermia (total motility 4.8 and 8.7%, progressive motility 0.6 and 0.4%, for the two patients) [56]. Transmission and scanning electron microscopy analyses and immunostaining for SLC26A8 and SEPT4 showed that the annulus was lost and the mitochondrial sheath was almost entirely absent in these patients. SLC26A8 may therefore play a more extensive role in mitochondrial anchoring in humans. Moreover, this study also provides interesting genetic information: the fathers of these two patients are heterozygous for the two missense mutations, indicating that these variants have no effect on fertility in the heterozygous state. However, the presence of two missense mutations in two different positions in one of the patients was sufficient to cause infertility and a loss of SLC26A8 [56]. These elements again highlight the genetic complexity and the importance of considering each mutation independently.

### SEPT4 mutations and human infertility

In mice, *Sept4* knockout results in a severe phenotype. Surprisingly, until recently, no *SEPT4* mutation had been identified in humans. Homozygosity for loss-of-function mutations of the *SEPT4* gene was eventually detected in two patients in 2022. The mutations (*c.A721T* (p.R241*) and *c.C205T* (p.R69*)) lead to a premature stop codon in exon 1 and underlie asthenoteratozoospermia [57]. As reported in the *Sept4* KO mouse [6], the annulus is completely lost and the MP is thin. The annular localization of SLC26A8 and of all the septins studied was also lost. However, only 20% of spermatozoa were folded at the MP/PP junction, demonstrating that the complete loss of the annulus does not systematically lead to the bending of the flagella in humans. Consequently, the human patients presented relatively moderate asthenozoospermia (35.4 and 45.3% total motility and 3.4 to 8.9% progressive motility).

### Comparison between human and mouse phenotypes

The phenotypic differences between mutations in humans and mice are of interest. Indeed, *SLC26A8* mutation leads to complete loss of the annulus in humans, whereas, in mice, the annulus persists but is abnormal and detached from the membrane [8, 56]. This may indicate slight differences in the role of SLC26A8 between the two species. The *SLC26A8* mutation in humans leads to a loss of septin localization at the annulus, consistent with the lack of a visible annulus on TEM. However, SEPT4 and SEPT12 seem to colocalize at the sperm neck. The authors hypothesized that SLC26A8 might not be directly involved in the assembly of septins into octameric filaments, instead being crucial for the organization of the MP and the relocalization of the annulus. However, it is also possible that septins cannot form an annulus in the absence of SLC26A8 in humans, and that they accumulate in the cytoplasm of the neck region if they remain unused. In the absence of a visible ring in patients with *SLC26A8* mutations, further studies will be required to determine the possible role of SLC26A8 in establishing or maintaining the annulus in addition to determining its location.

*SEPT4* mutation systematically leads to the loss of the annulus in both humans and mice, but it has a much milder impact on sperm motility in humans. Indeed, in humans, as described previously, total motility is 35 to 45% [57], while in mice we observe a complete lack of motility [6].

Finally, the overall architecture and structure of the MP is maintained in the *Sept4*^*−/−*^ and *Slc26a8*^*−/−*^ mouse models, whereas the mitochondria are completely disorganized or almost entirely absent in humans with mutations of this gene. This may indicate a more important role for the annulus, in the establishment and organization of the MP, in humans. These findings are important considering the differences (shape and size) of the mitochondrial sheaths between humans and mice. Moreover, loss of the annulus in humans has different consequences for sperm motility depending on whether the underlying cause is *SEPT4* or *SLC26A8* mutation. This suggests an additional annulus-independent role of SLC26A8 in motility. However, this result is difficult to interpret because the authors also reported the loss of SLC26A8 in the sperm of patients with *SEPT4* mutation. More detailed studies are required to improve the characterization of the differences in the role of the annulus and its protein composition between humans and mice.

## Concluding remarks

Studies over the last 10 years have identified a significant repertoire of proteins present at the annulus, but this structure remains enigmatic. Its intriguing dynamics during spermiogenesis make it difficult to study. In addition, this phenomena of annulus migration is furtive, difficult to visualize in vivo and cannot be reproduced in vitro. Despite the intensive work carried out in the different atlases, there remain inconsistencies concerning the precise timing of the appearance of flagellar growth during spermiogenesis [1] (Fig. 3). TEM only gives a frozen cross-sectional image at a precise moment and only in 2 dimensions. The cross-sectional analysis of the highly overlapping stages within the seminiferous tubes makes the analysis very difficult. New techniques (particularly in 3D) will be necessary to be able to precisely determine the stages of appearance of the different components of the flagellum.

The study of mouse models with mutations, knock-ins or knockouts of the genes encoding annulus proteins has revealed the importance of this structure in male fertility. Annulus abnormalities cause characteristic morphological defects, with the sperm bending into a hairpin at the MP/PP junction. Furthermore, the significant impact on the organization of the mitochondrial sheath and even on mitochondrial architecture observed suggests a potential role of the annulus in the establishment/integrity of these organelles. Additional studies and new technological approaches will be required to characterize the role of the annulus in these processes during spermiogenesis.

The role of the annulus does not seem to be limited to cytoskeletal functions. Indeed, this structure seems to be implicated in capacitation, a process involving numerous cellular signaling cascades. This result is not surprising given that septin polymers, the main constituents of the annulus, are known to act as “signaling platforms” in other cell models. During budding in yeast or cytokinesis in mammalian cells, septin filaments function as true multimolecular scaffolds capable of recruiting the components of signaling pathways [58, 59]. In particular, they are regularly found in association with kinases [60]. Septins are also capable of affecting the dynamics and organization of other cytoskeletal structures, including microtubules (in mammals [61, 62]) and actin (in *Drosophila* [63]). These elements highlight the possible role of the annulus in functional regulatory processes, such as capacitation, during sperm maturation, but also suggest possible involvement in spermiogenesis.

Finally, although few cases have been identified to date, recent studies have also highlighted the importance of the annulus for sperm physiology in humans. Annulus defects cause asthenoteratozoospermia, leading to infertility in men. However, the morphological defects visible by light microscopy are subtle and far removed from the severe defects observed in MMAF (multiple morphological abnormalities of the flagella for instance – [64]). Thinning of the MP has been demonstrated but the bending of the flagella (a characteristic feature of mouse models) is not systematically observed, possibly due to the small size of the MP in humans. It is likely that thinning of MP is counted in the “irregular caliber” category of semen analyses, more specifically, in the morphological description of the flagellum. Further improvements in diagnosis and in the investigation of these subtle defects will probably require a new categorization of defects during spermogram analysis.

## Data Availability

No datasets were generated or analysed during the current study.

## Abbreviations

ADGB: Androglobin
CYP24A1: 1,25-Dihydroxyvitamin D(3) 24-hydroxylase
GBD: GTB-binding domain
GM1: Ganglioside M1
KI: Knock-In
KO: Knockout
MMAF: Multiple Morphological Abnormalities of the Flagella
MP: Midpiece
NC: N- and C-termini
PKA: Protein Kinase A
PP: Principal Piece
PTM: Post-Translational Modifications
SACY: Soluble Adenylate Cyclase
SEPT: Septin
Spz: Spermatozoa
TAT1: Testis Anion Transporter
TEM: Transmission Electron Microscopy
